# Support System for Early Diagnosis of Chronic Obstructive Pulmonary Disease Based on the Service-Oriented Architecture Paradigm and Business Process Management Strategy: Development and Usability Survey Among Patients and Health Care Providers

**DOI:** 10.2196/17161

**Published:** 2020-03-17

**Authors:** Alberto De Ramón Fernández, Daniel Ruiz Fernández, Diego Marcos-Jorquera, Virgilio Gilart Iglesias

**Affiliations:** 1 Department of Computer Technology University of Alicante San Vicente del Raspeig, Alicante Spain

**Keywords:** COPD, misdiagnosis, business process management strategy, support system, service-oriented architecture, hospital information system, early diagnosis

## Abstract

**Background:**

Chronic obstructive pulmonary disease (COPD) is a chronic respiratory disease with a high global prevalence. The main scientific societies dedicated to the management of this disease have published clinical practice guidelines for quality practice. However, at present, there are important weaknesses in COPD diagnosis criteria that often lead to underdiagnosis or misdiagnosis.

**Objective:**

We sought to develop a new support system for COPD diagnosis. The system was designed to overcome the weaknesses detected in current guidelines with the goals of enabling early diagnosis, and improving the diagnostic accuracy and quality of care provided.

**Methods:**

We first analyzed the main clinical guidelines for COPD to detect weaknesses that exist in the current diagnostic process, and then proposed a redesign based on a business process management (BPM) strategy for its optimization. The BPM system acts as a backbone throughout the process of COPD diagnosis in this proposed approach. The newly developed support system was integrated into a health information system for validation of its use in a hospital environment. The system was qualitatively evaluated by experts (n=12) and patients (n=36).

**Results:**

Among the 12 experts, 10 (83%) positively evaluated our system with respect to increasing the speed for making the diagnosis, helping in interpreting results, and encouraging opportunistic diagnosis. With an overall rating of 4.29 on a 5-point scale, 27/36 (75%) of patients considered that the system was very useful in providing a warning about possible cases of COPD. The overall assessment of the system was 4.53 on a 5-point Likert scale with agreement to extend its use to all primary care centers.

**Conclusions:**

The proposed system provides a functional method to overcome the weaknesses detected in the current diagnostic process for COPD, which can help foster early diagnosis, while improving the diagnostic accuracy and quality of care provided.

## Introduction

### Chronic Obstructive Pulmonary Disease

Chronic diseases represent the leading cause of mortality worldwide, and are currently responsible for almost 60% of all deaths [1]. These diseases are of long duration and usually of slow progression, significantly affecting quality of life. In particular, cancer, diabetes, and heart and respiratory diseases are chronic diseases with relatively higher prevalence, and chronic obstructive pulmonary disease (COPD) is categorized in the latter group.

The Global Initiative for Chronic Obstructive Lung Disease describes COPD as “a common, preventable and treatable disease that is characterized by persistent respiratory symptoms and airflow limitation that is due to airway and/or alveolar abnormalities usually caused by significant exposure to noxious particles or gases” [2]. According to the latest Global Health Observatory data, COPD ranked as the third leading cause of death worldwide, responsible for approximately 5% of all deaths globally in 2015 (3.17 million deaths) [3]. The prevalence and burden of COPD are projected to increase over the coming decades due to the gradual aging of the population, cumulative smoking exposure, and an increase in underdiagnosis [4,5].

The main national and international scientific societies dedicated to the management of COPD developed consensus regulations and clinical practice guidelines, including recommendations for quality clinical practice [4,6-10]. However, the processes described in the current guidelines suffer from important weaknesses for each of the different subprocesses, especially in the diagnostic subprocess. Numerous studies have demonstrated that COPD is often misdiagnosed, leading to inappropriate treatments [11,12]. Accordingly, there has been substantial research effort dedicated to improving diagnosis based on the use of clinical decision support systems, mainly involving machine-learning techniques to make an accurate diagnosis [13-15]. However, since none of these initiatives is based on a standardized process, they have only offered partial solutions to resolving this problem.

Since COPD is a chronic disease, constant supervision is required throughout the patient’s life to obtain greater control and avoid possible exacerbations; thus, management of the disease imposes high costs to the health care system and population [7,16-19]. In this context, early diagnosis of COPD is essential to prevent its progression, improve the quality of life of patients, and reduce the economic impact borne by public health systems.

### Business Process Management

Fields such as manufacturing and business have long been developing strategies and paradigms related to process optimization for the continuous improvement of processes aimed at customer satisfaction and execution of these processes, resulting in models with demonstrated success. Business process management (BPM) is one of the most recent process management strategies with the greatest impact, which is focused on the continuous improvement of business processes using information technology as one of its fundamental principles for process execution [20]. BPM consists of a set of methodologies and technologies for the identification, modeling, analysis, execution, control, and improvement of business processes. The BPM strategy seeks to achieve flexibility and agility in the evolution and dynamism of business processes and their associated computer systems. Although the BPM strategy was initially applied in the health field for improvement of administrative tasks, in the last decade, such strategies and associated BPM systems (BPMSs) have begun to be used for the partial management of clinical processes [18,21-24] with successful outcomes. A BPMS includes tools for process modeling such as BPM notation (BPMN), which is a standard notation based on flowcharts that facilitate process modeling. This notation has been specifically designed to coordinate the sequence of processes and messages that flow between the participants for different activities [25]. BPMN provides a common language so that all parties involved can communicate the processes clearly, completely, and efficiently. For any BPMS, the BPMN can be directly translated into a format that best supports process execution.

In this study, we developed a support system for the early diagnosis of COPD based on the BPM approach, which was designed to foster early diagnosis, and improve diagnostic accuracy and the quality of care provided. These objectives will also help to indirectly achieve other secondary goals such as shortening diagnostic times, avoiding unnecessary visits to health care centers, creating awareness about the disease, and helping to reduce associated costs.

We first analyzed the current diagnostic process to identify its main weaknesses, and developed a redesign for its optimization. We then conducted a questionnaire-based survey in a hospital setting with experts and patients for a qualitative assessment of the architecture of the proposed support system and the developed prototype.

## Methods

### Current Chronic Obstructive Pulmonary Disease Diagnostic Process Analysis

**Figure 1:**
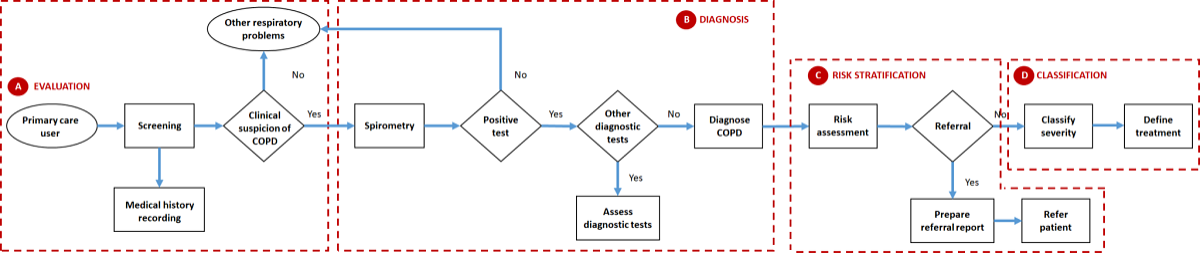
COPD diagnostic process in a primary care center.

The consensus regulations and clinical practice guidelines of the main national and international scientific societies include recommendations for quality clinical practice in the management of COPD [4,6-10]. In general, the diagnostic process for COPD is based on 4 steps (Figure 1): evaluation (Figure 1A), diagnosis (Figure 1B), risk stratification (Figure 1C), and classification (Figure 1D).

The current process begins in primary care centers when the patient is referred because of respiratory symptoms. The doctor is responsible for carrying out an initial screening to detect key indicators for considering a diagnosis of COPD. Clinical suspicion is recommended for any patient older than 35-40 years who has symptoms such as dyspnea, chronic cough, chronic sputum production, recurrent lower respiratory tract infections, exposure to risk factors, or a family history of COPD.

The doctor can perform a physical examination to detect, for example, edema or anomalous values of blood pressure, or respiratory and cardiac frequency. However, this test will rarely have diagnostic value, since the numerous physical signs of COPD typically do not appear until there is significant lung function impairment. In the case of clinical suspicion, the doctor must prepare a detailed medical history including information such as the patient’s exposure to risk factors, medical history of COPD or other chronic respiratory diseases, history of exacerbations, or presence of comorbidities.

To confirm the diagnosis, a postbronchodilator forced spirometry test must be performed. Airflow obstruction is considered to exist if the quotient between the forced expiratory volume in the first second (FEV1) and the forced vital capacity (FVC) is lower than 0.7. In case of doubt, and mainly to rule out alternative diagnoses or establish the presence of comorbidities, the doctor may request other complementary diagnostic tests, which generally include an X-ray, chest tomography, blood analysis, and pulse oximetry. With all this information, the doctor must be able to diagnose the patient a priori. Otherwise, the patient must be referred to hospital care to be further evaluated by a specialist [4].

The next step is to assess the severity of the disease. Since COPD is a heterogeneous condition, no single measure can adequately assess disease severity in an individual. In general, the degree of affectation is estimated by taking into account the airflow limitation (measured through FEV1), number of exacerbations, and degree of dyspnea, which allows for determination of severity among four stages or levels: mild, moderate, severe, and very severe. If the patient is determined to be at an advanced stage of the disease, they must be referred to hospital care for treatment by a pulmonologist. Once diagnosed, the doctor will define a treatment that allows for reducing the symptomatology to improve the patient’s quality of life, reduce the frequency of exacerbations, and control disease progression.

### Identification of Main Weaknesses in the Diagnostic Process

The diagnosis of COPD in primary care centers has inefficiencies and weaknesses that directly affect the patients’ quality of life and raise the economic cost borne by public health systems. In general, the diagnostic process starts when the patient suffering from respiratory problems arrives for a consultation. However, an “opportunistic” search is more profitable in a primary care setting [4,8]; that is, a physician takes the opportunity to assess whether the patient is among the at-risk population when they arrive for a consultation for other reasons. This is partly due to the fact that a large number of patients with COPD can remain asymptomatic until reaching advanced stages of the disease, which increases the ratio of undiagnosed individuals [5].

Standardization of respiratory function tests is necessary to achieve early detection and secondary prevention of the disease, including identification of all affected individuals and assessment of the severity of each patient. However, there are currently numerous nonunified clinical guidelines with differences both in the initial screening and in the assessment of the severity of the disease, resulting in the use of different diagnostic criteria in different countries. Some of these guidelines suggest that any adult with a respiratory symptom is considered to be at risk for COPD [10,26,27], whereas others discard individuals under 35 [7,8] or 40 years [4,6]. Other guidelines subordinate the clinical suspicion to the condition of smoker or ex-smoker [8,9], or to the score obtained in a screening test [28].

Some clinical guidelines establish the stage of the disease based solely on the FEV1, which measures airway obstruction [9,10]. Other guidelines apply multidimensional indices [6,8] that have demonstrated proven risk predictive capacity [29,30] taking into account FEV1 in addition to factors such as dyspnea, number of exacerbations, body mass index, or even the cardiopulmonary response to exercise. However, some authors absolutely reject the use of such factors, arguing that they do not provide better prediction outcomes than FEV1 alone, and require more time to classify and several resources that are not available in all primary care centers [7].

Spirometry is considered to be the most reproducible, standardized, and objective way of measuring airflow limitation; however, this can also be an important weak point in the diagnostic process. In many cases, this test is not performed or the quality of the test is very low (it is performed inefficiently) for different reasons, including lack of an available spirometer, the technique is not known or there is no experienced staff to perform the test, the results are not interpreted correctly, technological errors (calibration errors), errors made by the patient, or the results are not recorded in the medical history. Moreover, the fixed value threshold of 0.7 suggested by the main clinical guidelines can lead to overdiagnosis, and therefore a variable threshold based on the age and sex of the patient has been proposed [31,32].

Misdiagnosis of COPD also occurs due to errors made in primary care. Some studies have shown that professionals in primary care centers make more diagnostic errors than specialists, mainly due to lack of awareness of the disease, which often leads to the criteria for hospital referral to be ignored or the associated reports not including all of the recommended information [33].

A further challenge in COPD diagnosis is the similar symptomatology to other diseases such as asthma, lung cancer, acute coronary syndrome, and ischemic heart disease [34-38]. Finally, misdiagnosis can occur owing to factors related to the characteristics of the patient. For example, women in general and asymptomatic patients are at higher risk of underdiagnosis, as well as overweight or obese patients. In addition, different ethnic groups have different lung capacities that can also lead to misdiagnosis [33].

**Figure 2:**
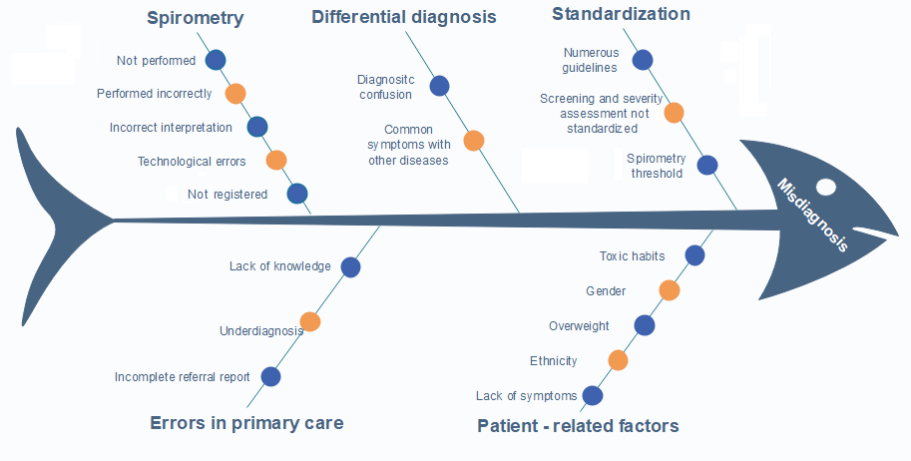
Ishikawa diagram of COPD misdiagnosis.

These inefficiencies result in substantial levels of misdiagnosis, underdiagnosis, and poor diagnostic accuracy. Some studies have shown that primary care physicians have problems in diagnosing up to 19.8% of patients with chronic respiratory symptoms and that the underdiagnosis rate exceeds 70% [12,39]. Figure 2 shows a cause-effect diagram based on Ishikawa et al [40] with a summary of the main weaknesses detected. All of these inefficiencies consequently result in increased costs associated with COPD diagnosis, mainly because the patients must return for several consultations until an accurate diagnosis is obtained, or due to overuse of hospital resources (diagnostic tests). Therefore, an early diagnosis would help to significantly reduce the costs associated with the treatment of a patient with COPD.

### Business Process Management Redesign Process

Based on the detected weaknesses described above, we here propose a redesign of the COPD diagnostic process to facilitate its comprehensive management and optimization. This redesign is based on a BPMS that serves as a backbone throughout the process and allows achievement of the main objectives: foster early diagnosis, improve diagnostic accuracy, and improve the quality of care provided. These objectives will be achieved from process standardization, the traceability of the tasks, notification to the patients, verification of the correct performance and correct interpretation of the spirometry test, diagnosis suggestion, and provision of all of the necessary information to assist the doctor during the diagnosis. The system functionalities are discussed below.

**Figure 3:**
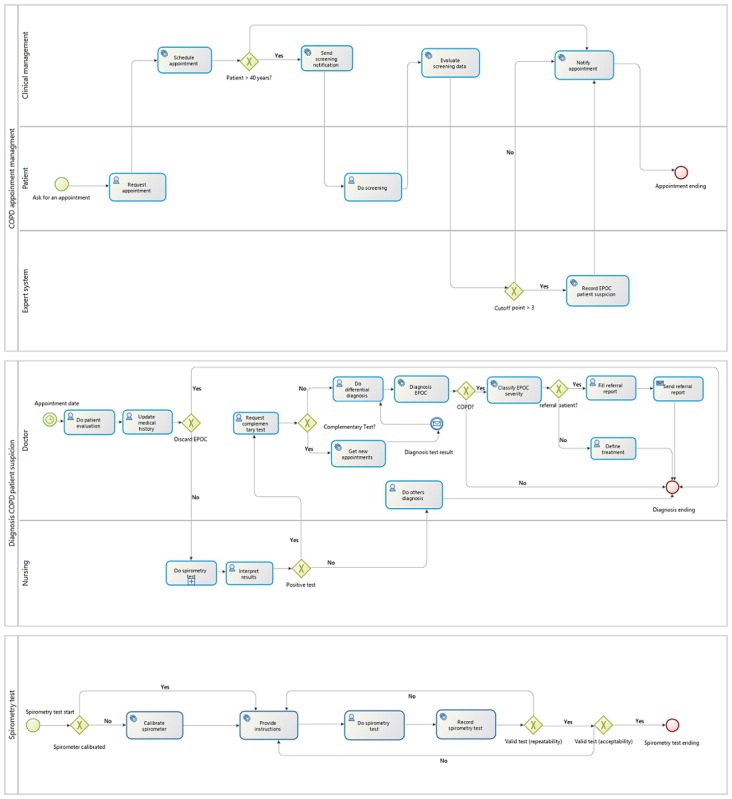
Chronic obstructive pulmonary disease diagnosis clinical process redesign using business process management notation. EPOC: excess postexercise oxygen consumption.

BPMS allows for modeling the process graphically using the BPMN that serves as a reference for standardization of the diagnosis, and to clearly define the tasks to be performed and the responsibility of the actors involved (doctor, nurse, patient, or the health information system [HIS]) in performing them (Figure 3). The modeling of the process allows the BPMS to ensure the traceability of all tasks and appropriate communication in real time to the professionals involved. The BPMS is responsible for controlling the process, guiding the professional, and ensuring that all of the tasks assigned are performed. Another important factor of a BPMS is the recording of the realization time of each task. This aspect is fundamental within a continuous improvement approach since it can help to detect which tasks are slowing/dilating the process or consuming more time, and the current process can be modified without the need to reprogram the entire system.

The process in the diagnosis of COPD begins at a preconsultation stage when a patient requests a medical appointment through an HIS. This appointment does not necessarily have to be motivated by a problem relating to COPD. The BPMS can interact with legacy systems, in this case, a hospital management system. If the patient requesting the appointment meets certain indications such as being over 40 years of age, the BPMS captures this information, classifies the patient within the population at risk, and sends a message to the patient so that they perform an initial screening (see Figure 3). This notification will only be sent to patients who have not already carried out this evaluation in the last 2 years. The screening allows for opportunistically detecting patients who are among the population at risk of COPD and, in many cases, in the early stages of the disease. The BPMS is responsible for collecting and evaluating the results of the screening. If the result is positive, both the patient and the doctor are notified of a probable case of COPD and the clinical suspicion is recorded in the system. In this way, the doctor knows the patient’s risk situation before the medical appointment. To prevent the patient from forgetting the appointment and improve the absenteeism rates of patients in primary care consultations, which have been estimated between 10% and 15% [38], the system sends a notification with a reminder 24 hours in advance of the appointment.

**Figure 4:**
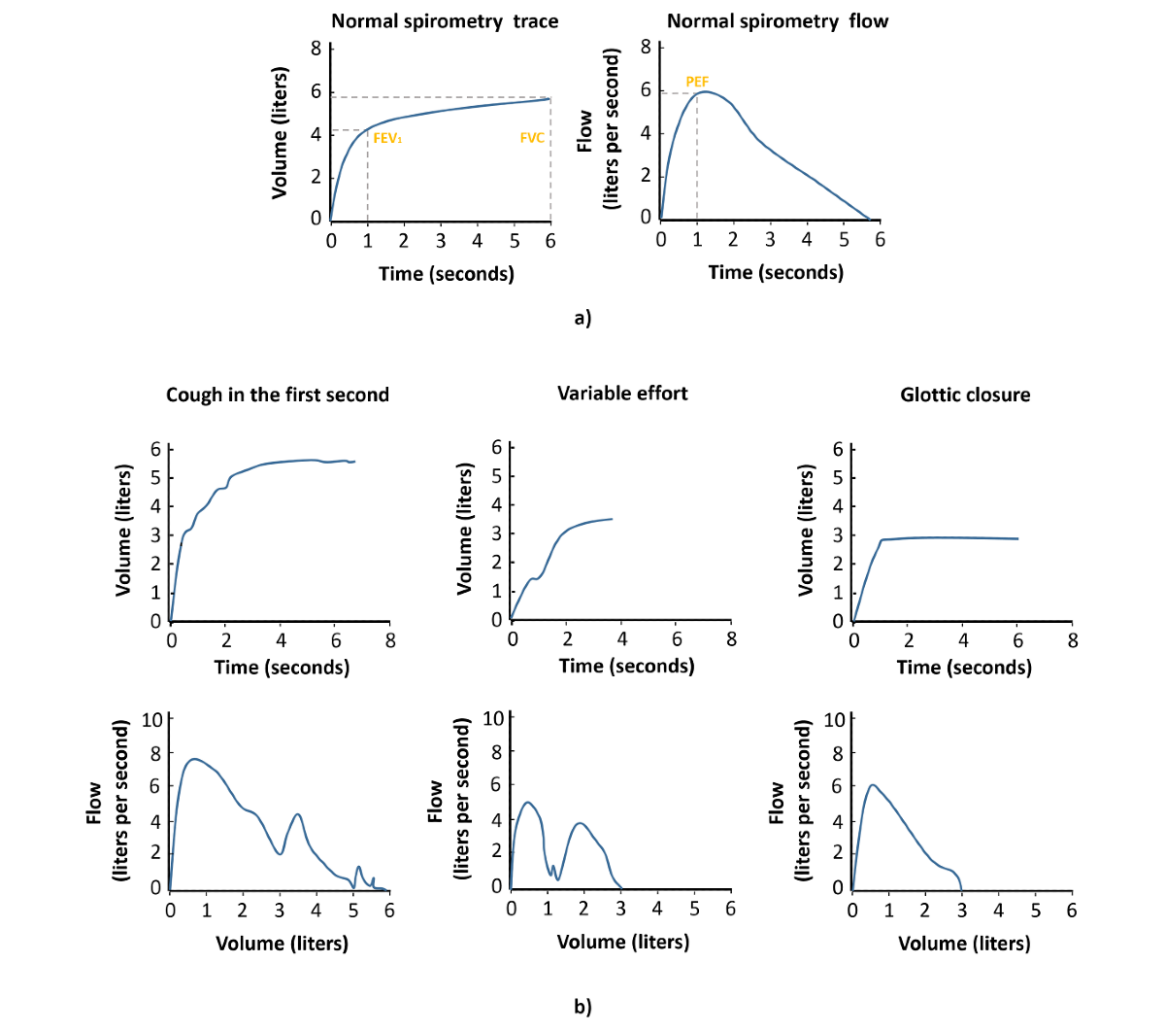
(a) Diagrams for normal spirometry; (b) Examples of incorrect spirometries. FEV1: forced expiratory volume (first second); FVC: forced vital capacity.

The process continues in the consultation on the day of the appointment with a more detailed evaluation of the patient by the doctor (symptomatology, risk factors, previous exacerbations, presence of comorbidities, family history, and impact on quality of life), along with an update of the patient’s medical history. If a case of COPD is suspected, the spirometry test is performed by the nurse. First, the BPMS is responsible for verifying if the spirometer is calibrated (usually by checking the last calibration date), and otherwise provides information and assistance to the professional for performing the calibration. Before performing the test, the system reproduces an audio file with instructions to the patient for the correct performance of the maneuver. The results are either collected automatically by the system or imputed manually by the professional, depending on the level of automation and integration of the spirometer. The test is supervised at all times by the nurse verifying its acceptability. To do this, the nurse must correctly interpret the spirometric diagrams shown by the spirometer. To assist the professional in this task, a template is shown (see Figure 4) with a normal spirometric volume flow curve and others incorrectly performed for different reasons (eg, slow start, early termination, glottis closure, or variable effort).

Next, the system checks whether the test meets the reproducibility criteria. For this step, the difference between the two best FVC and FEV1 values of the three attempts made must be less than or equal to 0.15 L. Otherwise, it is reported that the test is not valid and must be repeated. If both criteria are met, spirometry is validated and recorded, and the test result is analyzed based on the normal lower limit calculated for each patient according to their age, sex, and race. In this work, we have chosen to use this metric since some studies have shown that this approach has better diagnostic accuracy than the use of the fixed limit of 0.7 proposed by the main clinical guidelines [41,42]. If the spirometry result is negative, the patient is ruled out as having COPD and other diagnoses are assessed. In the case of a positive result, the doctor may require additional tests (eg, chest X-ray, blood count, oximetry) to confirm the diagnosis. With the results of the initial screening, medical history, spirometry results, and complementary tests, the doctor must make a differential diagnosis to rule out other respiratory diseases. This is a key step, since COPD may have common symptomatology to other diseases that induce diagnostic errors. At this point, the system provides key information on pathologies with a similar clinical picture to help the doctor make the differential diagnosis of COPD (see Table 1). Once other possible diagnoses have been ruled out, the doctor makes a diagnosis of COPD and proceeds to assess the severity of the disease.

**Table 1:** Differential diagnoses for respiratory diseases.

Diagnosis	Suggested Features
COPD^a^	Onset in midlife Symptoms slowly progressive History of tobacco smoking or exposure to other types of smoke
Asthma	Onset early in life (often in childhood) Symptoms vary widely from day to day Symptoms worse at night/early morning Allergy, rhinitis, and/or eczema also present Family history of asthma Obesity coexistence
Congestive heart failure	Chest X-ray shows dilated heart, pulmonary edema Pulmonary function tests indicate volume restriction, not airflow limitation
Bronchiectasis	Large volumes of purulent sputum Commonly associated with bacterial infection Chest radiograph/CT^b^ scans show bronchial dilatation, bronchial wall thickening
Tuberculosis	Onset at all ages Chest X-ray shows lung infiltrate Microbiological confirmation High local prevalence of tuberculosis
Obliterative bronchiolitis	Onset at younger age, nonsmokers May have history of rheumatoid arthritis or acute fume exposure Seen after lung or bone marrow transplantation CT on expiration shows hypodense areas
Diffuse panbrochiolitis	Predominantly seen in patients of Asian descent Most patients are male and nonsmokers Almost all cases involve chronic sinusitis Chest X-ray and HRCT^c^ show diffuse small centrilobular nodular opacities and hyperinflation

^a^COPD: chronic obstructive pulmonary disease.

^b^CT: computed tomography.

^c^HRCT: high-resolution computed tomography.

Classification of the severity of the disease is based on the automatic calculation of the exacerbation of the body mass index, airflow obstruction, dyspnea, and exercise (BODEx) [43] and/or (BODE) [44] indices together with evaluation of the impact of the disease on the patient's quality of life. To do this, together with the FEV1 value obtained in the spirometry, the body mass index, degree of dyspnea, number of exacerbations, and scoring of the 6-minute walk test (if applicable) and the COPD assessment test [39] are recorded.

The system classifies the patient according to four levels of severity (mild, moderate, severe, and very severe) and four levels of impact on their quality of life (low, moderate, high, and very high), and refers the case to a specialist if the degree of affectation is severe or very severe, or if frequent exacerbations are noted. In this case, the doctor must complete the referral report proposed by the system. Otherwise, the doctor defines the most appropriate treatment and ends the process.

### Evaluation

The experimentation phase of the proposal was approached from two different but complementary perspectives corresponding to the two types of users involved in the system: the health professionals who are the users and managers of the system, and the patients who are the direct beneficiaries of the execution of the proposed system. It is important to emphasize that both types of users are active users; that is, they are both involved and affected by the execution of the system. Therefore, we consider that the opinion of both types of users is the best result of experimentation that can be obtained, since validation with clinical experience would correspond to a project focused on clinical validation and not on the tool itself as in the present case.

To validate the achievement of the objectives proposed in the design of the system, a Likert-type survey [45] was conducted with 16 questions focused on the objectives associated with the project for the health care professional, and another survey of the same type with 6 targeted questions on the perception of quality of care and empowerment aimed at patients. Each question is associated with a numerical value from 1 to 5, with 1 being “completely disagree” and 5 being “totally agree.” In addition, another open question was incorporated so that the respondents can assess the system qualitatively. It was emphasized to the respondents to focus on evaluating the system comprehensively and not on its separate parts. The questions were designed so as to cover the objectives proposed in this work to provide a tool that can offer an idea of ​​the degree of achievement of the objectives. The issues raised in the questionnaire were related to the main objectives of promoting early diagnosis, improving diagnostic accuracy, and improving quality of care.

## Results

### System Architecture and Prototype

To validate the proposed redesign for COPD diagnosis, a technical architecture was developed that allows for the design of realistic systems. A prototype of the architecture was developed to demonstrate its suitability. Specific technological tools were selected for the prototype, although these could be replaced by similar tools provided they are in line with the architecture. 

Since this is a distributed architecture, in which different and geographically distributed users (patients and professionals), apps, and software modules are identified, we adopted a service-oriented architecture (SOA) that allows for proper integration of these aspects. Specifically, we selected RESTFul type services, which provide characteristics that are fundamental to the proposal such as reusability, scalability, low coupling, interoperability, and security [46]. 

**Figure 5:**
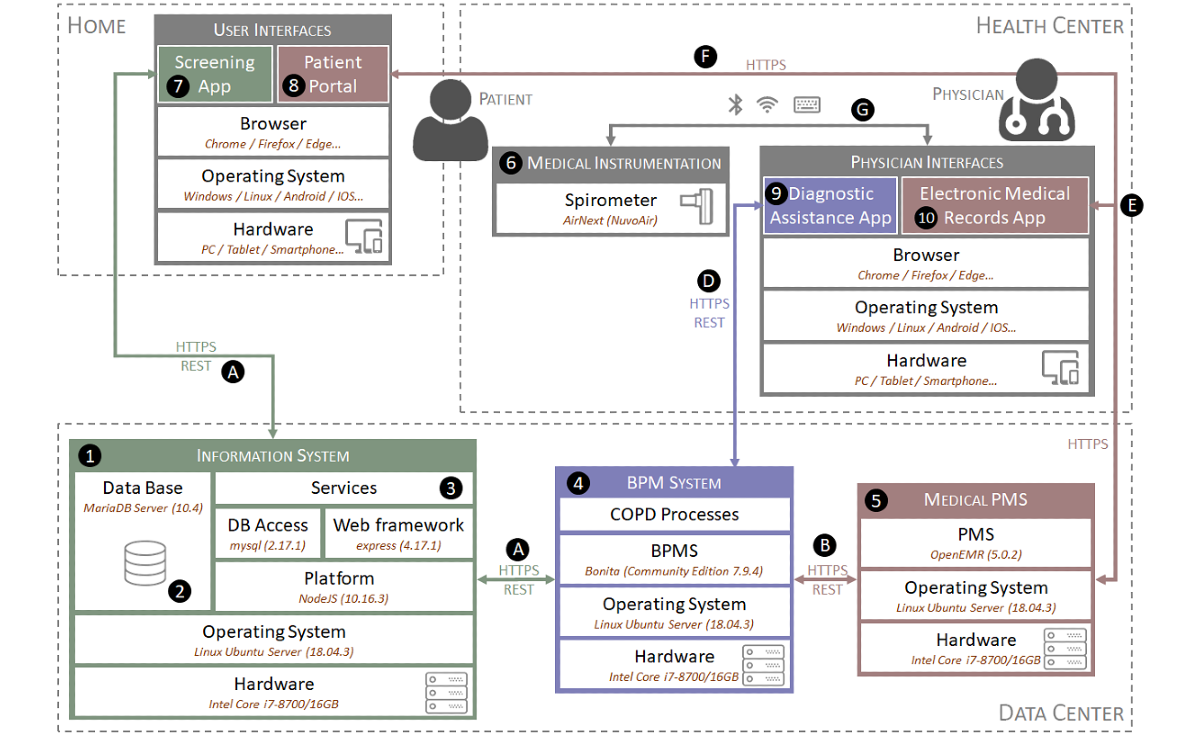
Proposed architecture and prototype. COPD: chronic obstructive pulmonary disorder; BPM: business process management; PMS: process management strategy.

**Figure 6:**
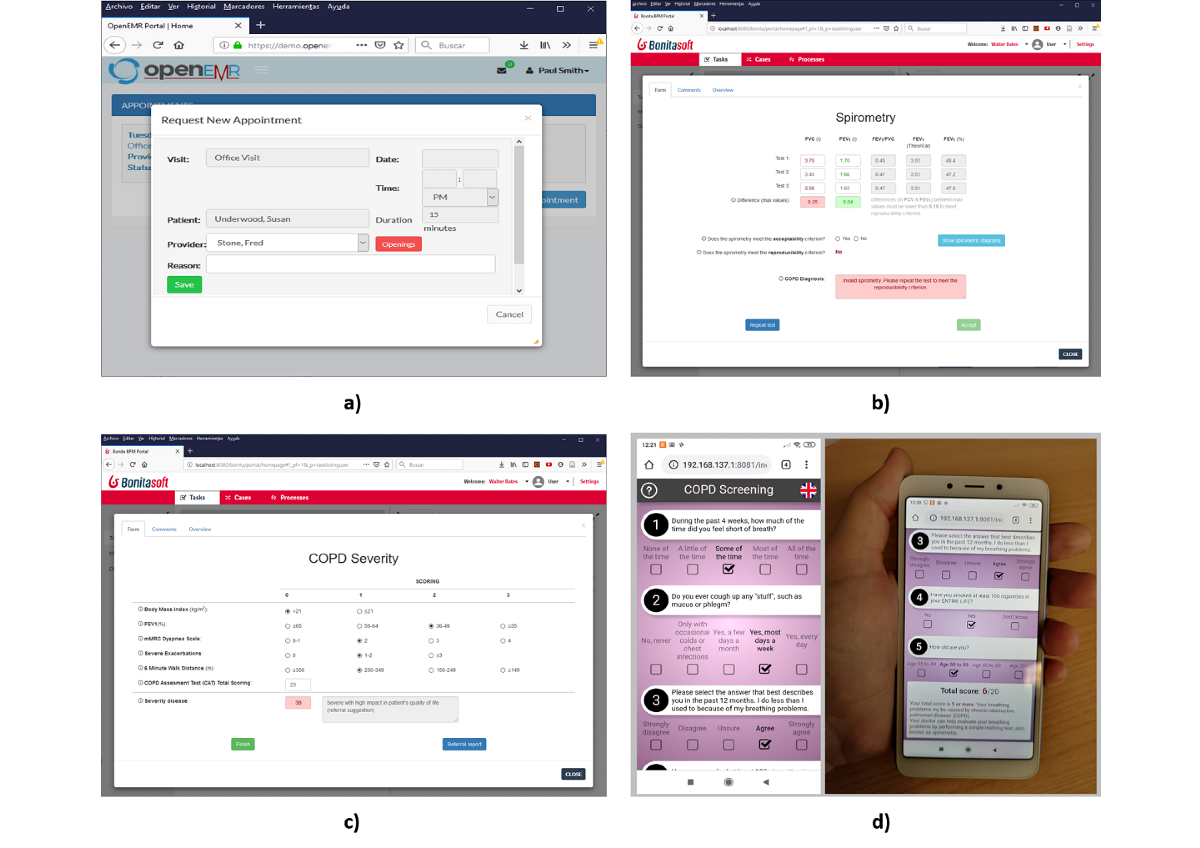
Prototype screenshots. (a) Appointment management from the OpenEMR Patient Portal. (b) Test spirometry interface. (c) Severity assessment interface. (d) Screening app.

The designed architecture (Figure 5) incorporates the following components as key points: 

- An information system (1 in Figure 5), which includes all of the necessary information for the proposed system. The information system is composed of two elements, a database server and an application server, that were run on a single device in the prototype but could also be implemented on different computers. 
- A database server (2 in Figure 5), in which all data necessary for the system will be saved. In the prototype, MariaDB is used as the database manager, which is a fork opensource of the MySQL relational database [47]. 
- An application server that implements a Representational State Transfer (REST) application programming interface (API) [48] (A in Figure 5) for access to the information system (3 in Figure 5). In the prototype, this server was developed using NodeJS as a platform together with the Express module for the development of HTTP services, and the MySQL module was used for access to the database. 
- A BPMS to execute the processes defined in the proposed redesign (4 in Figure 5). In the prototype, Bonita Software Community Edition (version 7.9.4; Bonitasoft, San Francisco, CA, USA) was used that allows access through a REST API (D in Figure 5). Toward this end, several conductors were developed for the different tasks identified in the process (Figure 3) that allow access to other elements of the architecture. 
- An electronic health records system (EHRS), in which the developed system is integrated. This is a key aspect of the proposal since it allows validating its use in a real environment (5 in Figure 5). For this purpose, we selected OpenEMR [49], which is a widely used open-source EHRS. This system uses a REST API (B in Figure 5) for access to its information (eg, patients, appointments, treatments). This API does not contain any notification mechanism; thus, the BPMS is not able to know when an appointment is requested by the patient. To solve this problem, at the end of each day, all of the new appointments made are consulted, those meeting the established criteria are analyzed, and, if applicable, a notification is sent to the patient to perform the screening. The following services are used for this process: auth (for authorization), appointment (for access to appointments), and patient (for access to patient data). 
- A spirometer (6 in Figure 5) with which the doctor can perform spirometry to the patient in consultation. This may be connected to the system or can operate independently, in which case the doctor must enter the data into the system manually (G in Figure 5). An AirNext model spirometer (NuvoAir AB, Stockholm, Sweden) was used in the prototype.
- A mobile app for the patient to perform the screening on COPD (7 in Figure 5 and Figure 6d). This app is operated by an app server that collects the test results using the defined REST API (C in Figure 5).
- The app that the patient uses to request the initial appointment (8 in Figure 5) that is the trigger of the COPD diagnosis process. In the prototype, the OpenEMR - Patient Portal app was used for this purpose (Figure 6a). 
- An app to assist the doctor in the diagnostic process (9 in Figure 5). For the prototype, an app was designed using Bonita Software forms (Figures 6b and 6c) that use the Bonita API (D in Figure 5). 
- The app with which the doctor manages the clinical process (10 in Figure 5). The Web app provided by OpenEMR was used in the prototype.

After development of the prototype, its functional validation was conducted, including complete cycles of COPD diagnoses from the request for an appointment by a patient to the final diagnosis by a professional.

### Evaluation Outcomes

Table 2 shows a list of the questions asked to the clinical staff, as well as the mean and median obtained for each question from the 5-point Likert scale.

**Table 2:** Questionnaire and result from the clinical perspective (N=12).

Questions	Mean (SD)	Median
Q1. The system will facilitate increasing the detection of COPD^a^ cases by opportunistic diagnosis	4.56 (0.51)	5
Q2. The system will allow improvements in COPD diagnosis	4.01 (0.79)	4
Q3. Thanks to the system, the patient will be aware of their pathology	3.24 (0.77)	4
Q4. The system will help avoid diagnostic confusion	3.09 (0.71)	3
Q5. The system will correctly assist health personnel in the diagnosis of COPD	4.31 (0.49)	4
Q6. The use of the system will help to avoid mistakes made in primary care centers due to lack of specialized personnel	4.47 (0.52)	5
Q7. The system will avoid unnecessary visits by patients with COPD	4.64 (0.49)	5
Q8. The system will help to standardize a protocol for the diagnosis of the disease	2.29 (1.00)	3
Q9. Thanks to the system, the interpretation of the spirometric test results will be improved	4.26 (0.77)	5
Q10. The use of the system will improve the coordination, monitoring, and traceability of the diagnostic process	2.45 (0.98)	3
Q11. The system will help speed up the diagnostic process	4.64 (0.49)	5
Q12. The system will improve the information provided by primary care centers to hospital centers	3.30 (0.49)	3
Q13. The system will help assess alternatives in the diagnosis	3.36 (1.00)	4
Q14. The use of the system will reduce the number of visits due to issues associated with COPD	4.31 (0.49)	4
Q15. The system will help improve spirometry performance	4.64 (0.49)	5
Q16. Overall assessment of the system	4.29 (0.65)	4

^a^COPD: chronic obstructive pulmonary disease.

The questionnaire was completed by 12 medical professionals, including family doctors (n=6) who monitor patients with COPD, emergency doctors (n=2) who care for patients when they have exacerbations, and nursing staff (n=4) who perform diagnostic tests. As shown in Table 2, the overall assessment of the system was generally good, highlighting the issues from Q7, Q11, and Q15 with mean scores above 4.5, indicating that health personnel strongly agree that the system can avoid unnecessary visits by patients with COPD, expedite the diagnostic process, and help improve the performance of spirometry. By contrast, the health personnel did not generally agree that the system would help to standardize a protocol for the diagnosis of the disease or that it would contribute to improving patient adherence to treatments. In this sense, it is important to highlight that these objectives would not be among the main associates of the system. In qualitative assessment, all respondents agreed on the need to use information and communications technologies to improve the quality of care, and this system is an example of this. Overall, 10/12 (83%) of the experts positively evaluated our tool to help speed up the diagnosis, help interpret the results, and also encourage opportunistic diagnosis, recommending its use in health centers, with an overall assessment of the system of 4.29 out of 5.

Table 3 shows the questions provided in the survey to the patients as well as the mean and median of their answers. The survey was completed by 36 patients with suspected COPD between 40 and 65 years of age, including 20 (55%) men and 16 (45%) women. Although this is a small sample, it serves as a proof of concept to present the overall perspective of patients regarding the benefits of the system. In general, the patients had a positive opinion about the use of the system; 27/36 (75%) of the patients considered that the system was very useful to warn about possible cases of COPD (Q1), highlighting their interest in the system being extended to different health centers (Q5). By contrast, patients did not view the system as a key factor in improving their awareness of the disease (Q4), matching with the opinion of the clinical staff on this aspect. The patients provided a global rating of the system of 4.53 out of 5.

**Table 3:** Questionnaire and results from the patient perspective (N=36).

Questions	Mean (SD)	Median
Q1. The system can help detect the disease	4.42 (0.50)	4
Q2. You feel more confident in the diagnosis thanks to the system	4.28 (0.67)	4
Q3. The use of the system helps improve your quality of life as a patient	4.04 (0.74)	4
Q4. The system helps you to be more aware of your disease	3.16 (0.63)	3
Q5. The use of the system should be extended to all health centers	4.76 (0.42)	5
Q6. Overall assessment of the system	4.53 (0.50)	5

## Discussion

### Principal Findings

COPD is a chronic respiratory disease that is associated with high morbidity worldwide. The main scientific societies dedicated to the care of patients with COPD have proposed different clinical guidelines over the years to help with its diagnosis and subsequent treatment. However, the COPD diagnostic process continues to present important weaknesses that cause late diagnosis or misdiagnosis. This has a direct impact on patient quality of life and the cost borne by health systems.

In this work, a support system for COPD diagnosis based on the BPM paradigm was developed in order to foster early diagnosis, and to improve diagnostic accuracy and the quality of care provided. The BPM strategy pursues, among other objectives, the optimization and standardization of processes, and allows the integration of human resources and information technology solutions through a BPMS that acts as a backbone throughout the diagnostic process.

To instantiate the redesign model raised for the diagnosis optimization, an architecture based on the SOA paradigm was designed that allows integration with characteristics of low coupling, reusability, scalability, interoperability, and security, typical of this type of architecture. The proposed architecture was designed to assist health care professionals during the diagnosis of COPD through the acquisition of patient information, storage and processing of data, and provision of the necessary clinical information for correct interpretation of the results.

From the definition of the architecture, a prototype was designed for a functional validation of the system. It is important to note that the system developed within an HIS was integrated into the prototype, which validates that the proposal can be integrated into a real hospital environment.

The system was also qualitatively validated by both clinical experts and patients. Overall, 83% of the experts surveyed positively evaluated our tool to help speed up the diagnosis, help interpret the results, and also encourage opportunistic diagnosis, recommending its use in health centers, with an overall assessment of the system of 4.29 on a 5-point scale. In addition, 75% of patients considered that the system was very useful to warn about possible cases of COPD, especially those who presented symptomatology compatible with the disease, and they agreed to extend its use to all primary care centers, with an overall assessment of the system of 4.53 on a 5-point scale.

### Limitations

This study has some limitations that must be addressed in the next steps of development. First, the implementation of external systems in a real hospital environment is quite restricted, which requires bureaucratic procedures and authorizations that affect different aspects of implementation. In addition, each hospital has its own hospital management system, and therefore it would be necessary to adapt our system to meet the integration requirements in each case. Second, and as a consequence of the first limitation, although the developed system overcomes the current weaknesses of guidelines for COPD diagnosis, it has not been possible to quantitatively validate how our system will improve the current diagnosis rates.

### Conclusions

This study highlights the difficulties that currently exist in the diagnosis of COPD in primary care centers. From a functional point of view, the proposed system can help to overcome the weaknesses detected in the current diagnostic process through integration of a mobile app so that patients can refer their symptoms, a spirometer to measure the patient’s lung capacity, and a Web app for physicians that allows them to consult all of the information provided by both the patients and the system. Implementation of this system is expected to help foster the early diagnosis, and improve the diagnostic accuracy and quality of care provided for COPD.

## Abbreviations

API: application programming interface
BODE: body mass index, obstruction, dyspnea, and exercise index
BODEx: exacerbation of body mass index, obstruction, dyspnea, and exercise index
BPM: business process management
BPMN: business process management notation
BPMS: business process management system
COPD: chronic obstructive pulmonary disease
EHRS: electronic health records system
FEV1: forced expiratory volume (first second)
FVC: forced vital capacity
HIS: health information system
REST: Representational State Transfer
SOA: service-oriented architecture
